# A case of an injured calcaneus secundarius in a professional soccer player

**DOI:** 10.1186/s12891-021-04246-0

**Published:** 2021-04-22

**Authors:** Kepka Sabrina, Morel Marc, Garnier Franck, Pietra François, Marjanovic Nicolas, Zeller Pascal, Bilbault Pascal, Kremer Stéphane, Bierry Guillaume

**Affiliations:** 1 Present Address: Emergency Department, University Hospital of Strasbourg, Nouvel Hôpital Civil, 1 place de l’Hôpital, CHRU, 67091 Strasbourg, France; 2 Medical Sports Center CMSM, Strasbourg, France; 3 Imaging Unit, Clinic Saint François, Haguenau, France; 4 School of osteopathy College COS Strasbourg - Franc Osteopathy Institute Research (IRFO), Strasbourg, France; 5 Emergency Department, University Hospital of Poitiers, Poitiers, France; 6 Imaging Unit 2, University Hospital of Strasbourg, Strasbourg, France

**Keywords:** Calcaneus secundarius, Soccer, Magnetic resonance imaging, Case report

## Abstract

**Background:**

The calcaneus secundarius (CS) is an accessory ossicle of the anterior facet of the calcaneus and is usually asymptomatic. This accessory bone can be frequently mistaken for a fracture of the anterior process of the calcaneus. Few reports of symptomatic CS have been published, and physicians need to be familiar with imaging strategies when encountering chronic ankle pain or in case of suspicion of fracture of the anterior process of the calcaneus.

**Case presentation:**

We describe the case of symptomatic CS in a professional soccer player injured during a match. First, computed tomography showed a large CS. Second, magnetic resonance imaging (MRI) demonstrated synchondrosis between the CS and the calcaneus, as well as edema (high MR T2 signal) within it, corresponding to posttraumatic edema. The patient was successfully treated with nonsteroidal anti-inflammatory drugs and physiotherapy; no surgical management was necessary. At the 4-week follow-up, he was pain-free and returned to activity.

**Conclusion:**

This case illustrates the role of imaging for the diagnosis of CS in cases of acute pain of the foot. CT, as well as MRI, helped to confirm the diagnosis of CS traumatized synchondrosis, which can be mistaken for a fracture.

## Background

The calcaneus secundarius (CS) is an accessory ossicle of the anterior facet of the calcaneus, usually asymptomatic, seen in up to 5% of the population [1, 2]. The CS is generally bridged to the calcaneus by poorly mobile synchondrosis. This accessory bone can be frequently mistaken for a fracture of the anterior process of the calcaneus in foot injuries or in persistent chronic ankle pain [3, 4]. Computed tomography (CT) or magnetic resonance imaging (MRI) might be indicated to assess the diagnosis. Few reports of symptomatic CS have been published [5–8], and physicians need to be familiar with imaging strategies when encountering chronic ankle pain or in cases of suspicion of fracture of the anterior process of the calcaneus. We describe the case of symptomatic CS in a professional soccer player injured during a match.

## Case presentation

A 26-year-old professional soccer player with no medical history presented with acute pain on the right foot consecutive to an injury during a match. The patient received a kick from an opponent on the outer face of the foot. During the initial examination by the team physician, there was moderate swelling of the soft tissue along the dorsal and lateral sides of the foot. No skin decoloration was present. On palpation, there was elective pain on the dorsal side of the foot. The testing of the midfoot revealed pain in the forefoot when walking or when pressure was applied, pain in push-off and unrolling of the foot, painful foot pronation and supination, and specific localized pain on the outer anteroside of the foot upon palpation. The patient was sent for imaging to rule out a fracture.

**Fig. 1:**
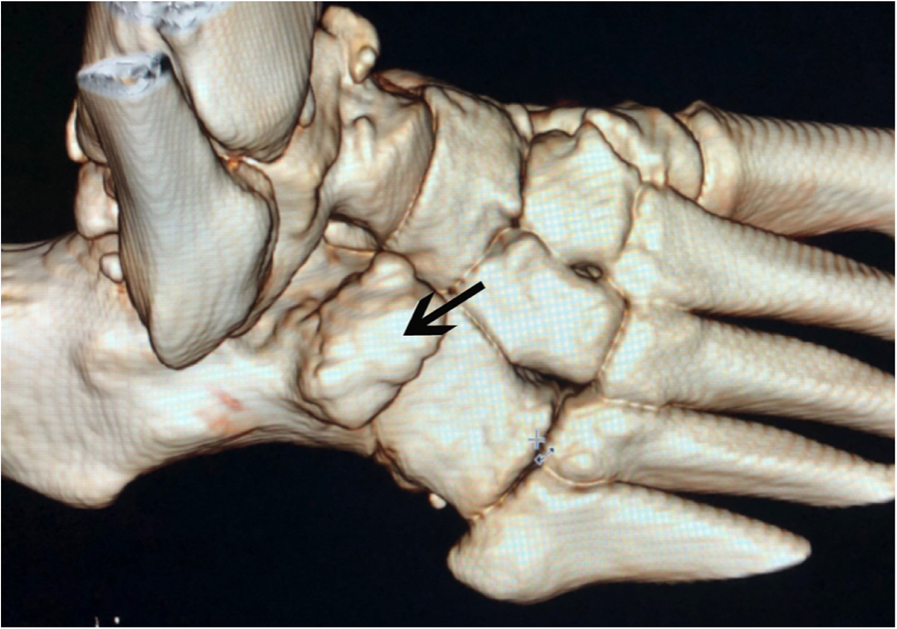
Three-dimensional volume-rendering CT image of the right foot. The arrow show the large calcaneus secondarius

**Fig. 2:**
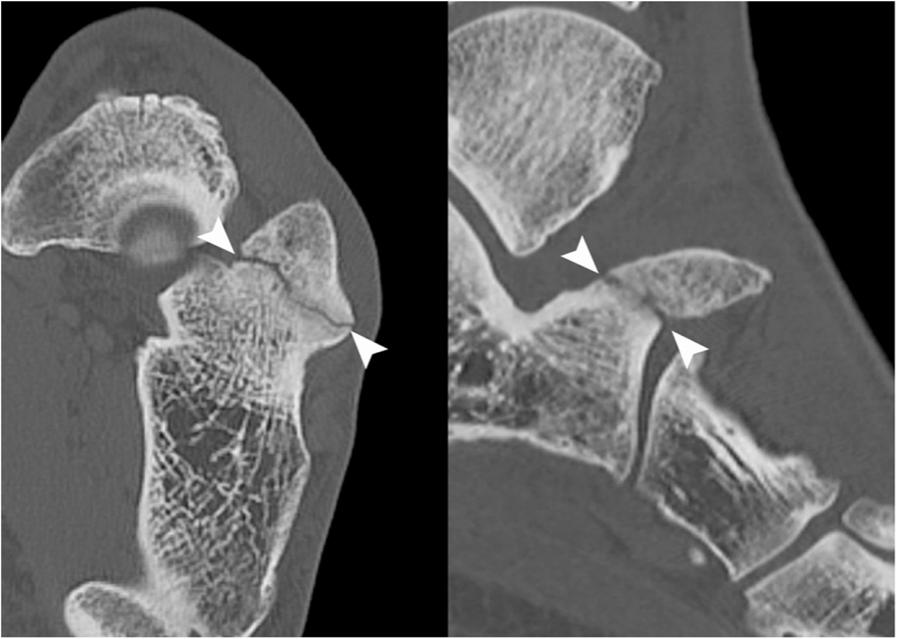
Axial (left) and sagittal (right) CT images. The arrowheads demonstrate the synchondrosis as a lucent line, that could be mistaken as a fracture

**Fig. 3:**
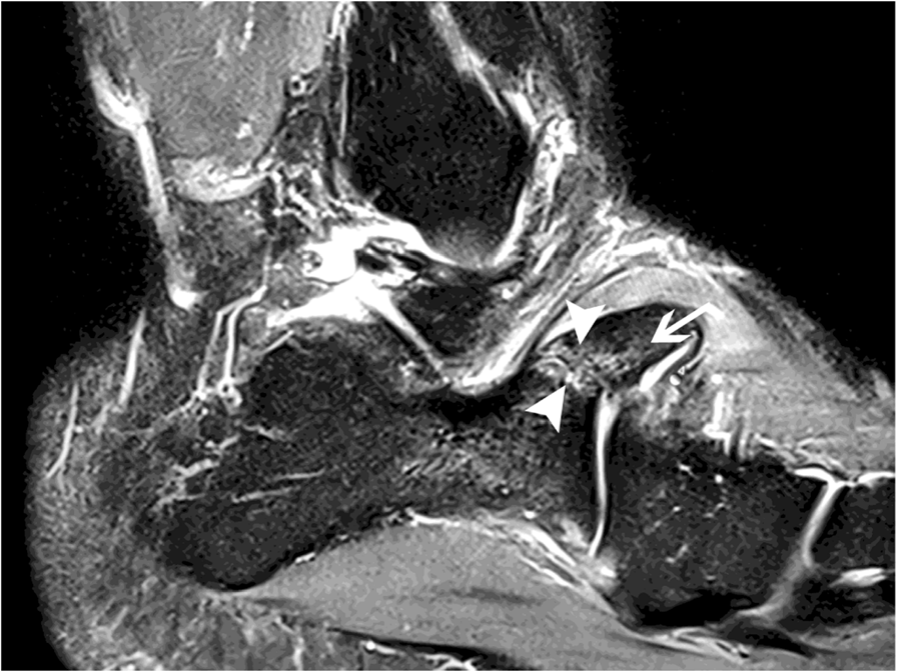
Sagittal T2-Fatsat MR image of the right foot. The arrow show the calcaneus secondarius with T2 hyperintensity corresponding to synchondrosis edema (arrowheads)

The professional status of this patient required diagnostic accuracy because it had an impact on his professional activity. The first diagnosis mentioned was a fracture, and the imaging strategy consisted of performing a CT of the foot. The calcaneus secundarius (CS) was observed as a large bony structure above the calcaneus anterior process (35 mm long, 30 mm high, 25 mm width) (Figs. 1 and 2)*.* Subsequently, a MRI was necessary to search for other impacts on the area, such as bone edema and other associated joint-type lesions, because it is the only imaging that can show associated bone suffering in the form of an edematory hypersignal on fat suppression sequences. First, MRI confirmed the diagnosis of CS by showing synchondrosis between the CS and the calcaneus, and second, MRI demonstrated posttraumatic edema within it as an area of increased T2 signal (Fig. 3).

Nonsurgical management consisted of nonsteroidal anti-inflammatory drugs and discharge for 7 days. We detailed the rehabilitation protocol in Table 1. The protocol presented here is a functional and conservative treatment tailored to one individual and accounting for the evolution of the pain. A physical therapy program of Scottish baths (alternating 4 cycles of 4 min of hot foot bath and 1 min of cold foot bath), bike and proprioception exercises was conducted for 4 weeks. Capacitive-resistive electric transfer therapy (Tecar) was also used by the physiotherapist. Walking with partial support was possible after 7 days, returning to running in a straight line and side support after 2 weeks. At 4 weeks of follow-up, he returned to training without strapping. No surgical management was necessary.

**Table 1:** Rehabilitation protocol

Period (days)	Activity	Tecar therapy	Physiotherapy	Treatments
0 to 3	Rest	Pulsed short Waves	Manual drainage	Phytotherapy (Porphyral HSP® and Arnica 9 CH®)
3 to 10	-Walking with partial support with crutches -Bike with a support on the heel - Core strength and fitness training of the upper body -Upper body cardio exercise		-Scottish baths -Manual drainage -Osteopathic treatment	
11 to 15	-The same program as J3 to J10 - Treadmill walking with respect for pain - Open chain muscle building of the entire lower body -Stop crutches at J15		-Strapping for activity -Manual drainage -Osteopathic treatment	Nonsteroidal anti-inflammatory 48 h
15 to 21	-Resumption of jogging on the football field with foot strapping -Athletic preparation oriented football with lateral support and change of pace at J18		-Massage -Osteopathic treatment -Joint mobilization -Scottish baths -Proprioception	
21 to 30	-Training session with strapping for 5 days then without strapping		-Manual drainage -Osteopathic treatment -Talocrural and subtalar joint mobilization -Scottish baths -Proprioception	

## Discussion and conclusion

Accessory ossicles are common in the foot and ankle, and 24 types of accessory ossicles have been reported in the literature [1]. These ossicles are usually asymptomatic and incidentally detected on radiographs. Nevertheless, they could be clinically relevant, especially in trauma, where they can be painful by themselves or simulate a fracture. Knowledge of the anatomy and presentation of these accessory ossicles might therefore be useful for physicians caring for injured patients.

The CS is an accessory ossicle of the anterior facet of the calcaneus, located between the anteromedial aspect of the calcaneus, the proximal aspect of the cuboid and navicular, and the head of the talus [1]. CS is seen in up to 5% of the population [1, 2]. CS is derived from the failure of union of secondary ossification centers and, like other accessory ossicles, it is generally bridged to the calcaneus by poorly mobile synchondrosis but can also be completely independent. The size of this ossicle is generally reported to be 3–4 mm in diameter [9]. One of the largest symptomatic CS was described in a 51-year-old man at 22 mm long and 16 mm high [3]. To our knowledge, the CS described in our case was the largest published so far (35 mm long, 30 mm high, 25 mm width). A sufficient size could cause deformity and/or limitations in the range of motion [10].

To date, only a few cases of symptomatic CS have been published, mostly in the setting of chronic ankle pain [4–7]. To our knowledge, no such presentation of an acutely injured large CS has been reported. The diagnosis of symptomatic CS can be challenging for emergency physicians because this accessory bone can easily be mistaken for a fracture of the anterior process or the tuberosity of the calcaneus [3, 4]. Moreover, it has to be differentiated from other accessory ossicles, such as a calcaneus accessorius, cuboideum secundarium, and os sustentaculi [1]. Indeed, the management of a painful CS is very distinct from the treatment of a calcaneus fracture, therefore requiring a well-adapted imaging strategy [11].

CS can sometimes be diagnosed on a lateral oblique view radiograph showing sclerotic and irregular margins of the calcaneus and cuboid adjacent to the bony fragment, suggestive of a chronic lesion [12]. Frequently, however, both physical examination and conventional radiographs are unable to differentiate a CS from a fracture, especially for emergency physicians not familiar with this presentation. Cross-sectional imaging, such as CT and MRI, is sometimes needed to distinguish CS from a fracture and to understand its clinical relevance. CT scans can confirm the diagnosis of an accessory bone by showing synchondrosis as smoothly and sharply margined well-corticated bones [4, 12]. MRI will confirm the diagnosis of synchondrosis and reveal its recent injury by showing diffuse edema [5, 7, 10].

No consensus exists on the management of an injured CS. Surgical excision of the ossicle is indicated if the symptoms do not resolve with conservative treatment or if the range of motion of the subtalar joint is limited [6–8]. Kraft et al. revealed the case of an injured CS in a 51-year-old man treated with an infiltration of steroids and local anesthetic keeping pain free for 1 month. However, the return of symptoms within several weeks requires surgical excision [8]. Furthermore, the case of a 54-year-old woman presenting a symptomatic injured CS that was initially conservatively managed has been reported. However, successful endoscopic resection was performed because she complained of persistent ankle pain [12]. We think that conservative treatment should be proposed, as shown in our case. Ersen et al. described the success of symptomatic therapy (nonsteroidal anti-inflammatory drugs and mobilization after 1 month) [5]. To our knowledge, there are no data in the literature regarding a rehabilitation protocol for symptomatic injured CS. The originality of this case lies in the precise description of a protocol for a conservative approach that has successfully supported a professional soccer player. This example could help clinicians in cases of painful injured CS by adapting the protocol to the pain and patient’s activity.

CS is a frequent cause of chronic ankle pain. However, the diagnosis of a painful injured CS can be considered in cases of acute ankle trauma in which a fracture of the anterior facet of the calcaneus is suspected. If the fracture seems atypical, with, for instance, an excessively large bone fragment or with corticalized borders, CT or MRI seems to be required to first distinguish synchondrosis from fracture and second to assess the acute component of the injury. Conservative treatment is recommended with a rehabilitation protocol based on physiotherapy, tecar therapy and gradual activity. Endoscopic resection should only be proposed in cases where this treatment fails.

## Data Availability

The datasets used and/or analysed during the current study are available from the corresponding author on reasonable request.

## Abbreviations

CS: Calcaneus secundarius
CT: Computed Tomography
MRI: Magnetic Resonance Imaging

## References

[1] Keles-Celik N, Kose O, Sekerci R, Aytac G, Turan A, Güler F (2017). Accessory Ossicles of the foot and ankle: disorders and a review of the literature. Cureus.

[2] Anderson T (1988). Calcaneus secundarius: an osteo-archaeological note. Am J Phys Anthropol.

[3] Hodge JC (1999). Anterior process fracture or calcaneus secundarius: a case report. J Emerg Med.

[4] Bulut MD, Yavuz A, Bora A, Gökalp MA, Özkaçmaz S, Batur A (2014). Three-dimensional CT findings of Os calcaneus Secundarius mimicking a fracture. Case Rep Radiol.

[5] Ersen O, Akyildiz F, Ozyurek S, Sivrioglu AK (2013). Os calcaneus secundarius mimicking fracture. BMJ Case Rep.

[6] Ceroni D, De Coulon G, Spadola L, De Rosa V, Kaelin A (2006). Calcaneus secundarius presenting as calcaneonavicular coalition: a case report. J Foot Ankle Surg.

[7] Stauss J, Connolly LP, Perez-Rossello J, Treves ST (2003). Skeletal Scintigraphy of possible Os calcaneus Secundarius. Clin Nucl Med.

[8] Krapf D, Krapf S, Wyss C (2015). Calcaneus secundarius--a relevant differential diagnosis in ankle pain: a case report and review of the literature. J Med Case Rep.

[9] Coughlin MJ, Mann RA, Saltzman CL (2007). Surgery of the foot and ankle.

[10] Krause JO, Rouse AM (1995). Accessory calcaneus: a case report and literature review. Foot Ankle Int.

[11] Kürklü M, Köse Ö, Yurttas Y, Ŏguz E, Atesalp AS (2010). Anterosuperior calcaneal process fracture or os calcaneus secundarius?. Am J Phys Med Reh.

[12] Lui TH (2018). Endoscopic resection of symptomatic os calcaneus secundarius. Foot..

