# Correction to: A case of an injured calcaneus secundarius in a professional soccer player

**DOI:** 10.1186/s12891-021-04343-0

**Published:** 2021-05-15

**Authors:** Sabrina Kepka, Marc Morel, Franck Garnier, François Pietra, Nicolas Marjanovic, Pascal Zeller, Pascal Bilbault, Stéphane Kremer, Guillaume Bierry

**Affiliations:** 1 Present Address: Emergency Department, University Hospital of Strasbourg, Nouvel Hôpital Civil, 1 place de l’Hôpital, CHRU, 67091 Strasbourg, France; 2 Medical Sports Center CMSM, Strasbourg, France; 3 Imaging Unit, Clinic Saint François, Haguenau, France; 4 School of osteopathy College COS Strasbourg - Franc Osteopathy Institute Research (IRFO), Strasbourg, France; 5 Emergency Department, University Hospital of Poitiers, Poitiers, France; 6 Imaging Unit 2, University Hospital of Strasbourg, Strasbourg, France

**Correction to:** ***BMC Musculoskelet Disord*** **22, 374 (2021)**

**https://doi.org/10.1186/s12891-021-04246-0**

Following the publication of the original article [1] the authors noticed a mistake in the author names. The surname and first name are inverted for all authors.

It was written as below.

Kepka Sabrina, Morel Marc, Garnier Franck, Pietra François, Marjanovic Nicolas, Zeller Pascal, Bilbault Pascal, Kremer Stéphane, Bierry Guillaume.

However, the correct order should be:

Sabrina Kepka, Marc Morel, Franck Garnier, François Pietra, Nicolas Marjanovic, Pascal Zeller, Pascal Bilbault, Stéphane Kremer, Guillaume Bierry.

The original article [1] has been updated.

## References

[1] Kepka S, Morel M, Garnier F (2021). A case of an injured calcaneus secundarius in a professional soccer player. BMC Musculoskelet Disord.

